# A new paradigm for depression in new mothers: the central role of inflammation and how breastfeeding and anti-inflammatory treatments protect maternal mental health

**DOI:** 10.1186/1746-4358-2-6

**Published:** 2007-03-30

**Authors:** Kathleen Kendall-Tackett

**Affiliations:** 1 Family Research Laboratory, 126 Horton Social Science Center, 20 College Road, University of New Hampshire, Durham, New Hampshire, 03824, USA

## Abstract

**Background:**

Research in the field of psychoneuroimmunology (PNI) has revealed that depression is associated with inflammation manifested by increased levels of proinflammatory cytokines.

**Discussion:**

The old paradigm described inflammation as simply one of many risk factors for depression. The new paradigm is based on more recent research that has indicated that physical and psychological stressors increase inflammation. These recent studies constitute an important shift in the depression paradigm: inflammation is not simply *a *risk factor; it is *the *risk factor that underlies all the others. Moreover, inflammation explains why psychosocial, behavioral and physical risk factors increase the risk of depression. This is true for depression in general and for postpartum depression in particular. Puerperal women are especially vulnerable to these effects because their levels of proinflammatory cytokines significantly increase during the last trimester of pregnancy – a time when they are also at high risk for depression. Moreover, common experiences of new motherhood, such as sleep disturbance, postpartum pain, and past or current psychological trauma, act as stressors that cause proinflammatory cytokine levels to rise. Breastfeeding has a protective effect on maternal mental health because it attenuates stress and modulates the inflammatory response. However, breastfeeding difficulties, such as nipple pain, can increase the risk of depression and must be addressed promptly.

**Conclusion:**

PNI research suggests two goals for the prevention and treatment of postpartum depression: reducing maternal stress and reducing inflammation. Breastfeeding and exercise reduce maternal stress and are protective of maternal mood. In addition, most current treatments for depression are anti-inflammatory. These include long-chain omega-3 fatty acids, cognitive therapy, St. John's wort, and conventional antidepressants.

## Review

Depression in new mothers is common in many cultures, affecting anywhere from 10% to 20% of postpartum women. In some high-risk populations, the percentage can even be as high as 40% or 50% [1]. Since depression has devastating effects on both mother and baby, it's vital that it be identified and treated promptly. Depressed mothers are also more likely to stop breastfeeding with negative health effects for each [1]. In this paper, I describe a psychoneuroimmunology (PNI) framework for depression in new mothers and discuss its implications for breastfeeding women.

### Inflammation and depression

In recent years, researchers in the field of PNI have found that inflammation is involved in the pathogenesis of depression. Maes and colleagues first documented that mothers with the postpartum blues had higher levels of inflammation than mothers who did not [2]. They concluded that the postpartum blues were caused by an activated inflammatory response system (IRS). In a later paper, Maes and colleagues noted that "it is generally accepted that in the early puerperium, there is an increased inflammatory responsivity in the serum ... suggesting an activation of the inflammatory response system" [3] (p. 71).

**Figure 1:**
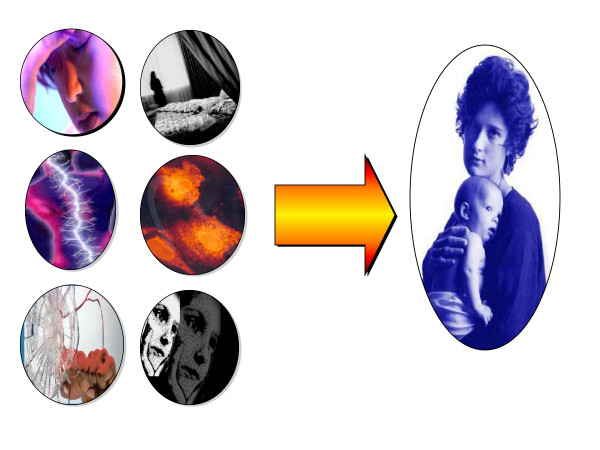
**Risk Factors for Depression in New Mothers – Old Paradigm**. depicts six risk identified risk factors for depression in new mothers: stress, sleep disturbance, pain, inflammation, psychological trauma, and a history of abuse depression or trauma.

When researchers first identified inflammation as increasing the risk of depression, most considered it an independent risk factor along with several others. The other risk factors included psychosocial stress, a broad category that included significant life changes, lack of social support, marital difficulties, infant illness or prematurity, and low income. Psychological trauma, another risk factor, includes current trauma or a history of trauma, which creates a vulnerability to future stressors even when there are no current symptoms. Sleep disturbance and pain are physical stressors that are common among new mothers and increase the risk of depression. The model portrayed on Figure 1 shows these as individual risk factors and represents the old paradigm.

**Figure 2:**
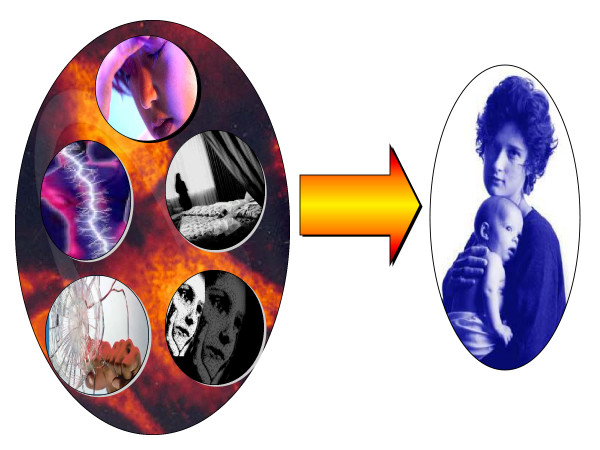
**Risk Factors for Depression in New Mothers – New Paradigm**. depicts five risk factors for depression in new mothers (stress, sleep disturbance, pain, psychological trauma and a history of abuse or trauma) with inflammation as the underlying risk factor.

More recent research, however, suggests a new paradigm for understanding depression. PNI researchers have found that physical and psychosocial stressors (i.e., all the risk factors for depression) increase inflammation [4-6]. These recent studies constitute an important shift in the depression paradigm: inflammation is not simply *a *risk factor; it is *the *risk factor that underlies all the others [7]. This model is portrayed on Figure 2.

Puerperal women are especially vulnerable because their inflammation levels rise significantly during the last trimester of pregnancy – a time when they are also at high risk for depression. Moreover, common experiences of new motherhood, such as sleep disturbance, postpartum pain, psychological stress, and trauma also increase inflammation. Physical and psychosocial risk factors for depression are still important to identify. The old paradigm identifies the known causes of depression in new mothers.

The new paradigm moves the field forward and provides an answer to the next question: why? Why would psychosocial risk factors, such as lack of social support or marital strife or stress, increase the risk of depression? Why would depression be more likely in women who are sleep deprived or who have experienced trauma or pain? The new paradigm provides perspective on the mechanism by which previously identified risk factors increase risk. It also guides practitioners in their intervention efforts and suggests that targeting inflammation may increase women's resilience to other stressors.

### Breastfeeding and depression in mothers

Breastfeeding also has an important role to play in mothers' postpartum mental health. Groër and Davis noted that "breastfeeding confers some psychoneuroimmunological benefits to mothers" (p. 599) in part because of its impact on stress [8]. In an earlier review, Groër, Davis and Hemphill noted that although women experience many stressors in the postpartum period, breastfeeding protects them by inducing calm, lessening maternal reactivity to stressors, and increasing nurturing behaviour [9]. The PNI approach is relevant to lactation specialists because it demonstrates that breastfeeding can protect mothers' mental health and is worth preserving whenever possible.

### Method of literature review

The studies cited below were assembled from a wide variety of sources. Literature searches on PubMed and PsychInfo were conducted on depression, postpartum/postnatal depression, depression and inflammation, proinflammatory cytokines, pain, sleep disturbances, HPA dysfunction and depression, trauma, omega-3 fatty acids, inflammation and depression. In addition, several key PNI and psychiatry journals were manually searched for relevant articles including *Psychoneuroendocrinology*, *Psychosomatic Medicine*, *Psychosomatic Research*, *Biological Psychiatry*, *Health Psychology*, *American Journal of Psychiatry*, *British Journal of Psychiatry*, and *British Medical Journal*. Where appropriate, literature from the field of cardiovascular medicine was cited as this literature contains many studies that examine the links between depression, inflammation and risk of cardiovascular disease.

### Physical and psychological stressors that increase inflammation and risk of depression

To understand the role of inflammation in depression, it's helpful to first review the normal physiologic response to stress. When faced with a threat, human bodies have a number of interdependent mechanisms in place designed to preserve our lives. The sympathetic nervous system responds by releasing catecholamines (norepinephrine, epinephrine, and dopamine). The hypothalamic-pituitary-adrenal (HPA) axis also responds: the hypothalamus releases corticotrophin releasing hormone (CRH), the pituitary releases adrenocorticotropin hormone (ACTH), and the adrenal cortex releases cortisol, a glucocorticoid. The immune system responds by increasing production of proinflammatory cytokines, which increase inflammation. Cytokines are proteins that regulate immune response. Proinflammatory cytokines help the body heal wounds and fight infection by stimulating an inflammatory response [10].

Maes and colleagues described the interrelatedness of these systems and noted that inflammation influences levels of serotonin and catecholamines, and has an impact on the HPA axis, which controls cortisol levels [7,11]. Once inflammation starts, it triggers the HPA axis to release cortisol, and the release of more proinflammatory substances [12]. Breastfeeding appears to attenuate these effects by lowering cortisol, ACTH, epinephrine and norepinephrine [8].

#### (1) Immune and HPA dysfunction in depression

Depression is also related to some distinct abnormalities in the immune system and HPA axis. I will describe the immune effects first. For many years, researchers considered depression to be primarily immunosuppressive in that they observed that depressed people had fewer lymphocytes and the natural killer cells (NK) had lower cytotoxicity [6,13]. These are both indications of a suppressed immune system. More recent studies, however, indicate that depression causes an immune dysfunction, meaning that some aspects are suppressed, while other aspects are elevated [13,14]. In depressed people, inflammation is increased, including high levels of proinflammatory cytokines and acute-phase proteins, such as C-reactive protein (CRP), which are a physiologic response to chronic distress [6,12,13]. Levels of inflammation can be 40% to 50% higher in depressed people than their non-depressed counterparts [15].

The proinflammatory cytokines that researchers identified most consistently as being elevated in depression are interleukin-1β (IL-1β), interleukin-6 (IL-6), tumor necrosis factor-α (TNF-α), and more recently, interferon-γ (IFN-γ) [15]. In the last trimester of pregnancy, levels of proinflammatory cytokines rise [2]. When these cytokines are within normal levels, they are adaptive because they help prevent infection. When they are abnormally high, they increase the risk of depression. Proinflammatory cytokines cause a constellation of sickness behaviors in humans, which includes alterations in sleep, appetite, activity, mood, energy, sexual activity and socialization – all behaviors associated with depression [2]. The relationship between inflammation and depression appears to be bidirectional: inflammation increases the risk of depression and depression increases inflammation.

Depression can also influence the systems that normally keep inflammation in check, such as the HPA axis. Cortisol is anti-inflammatory and is generally secreted when inflammation levels get too high. However, depressed people either have abnormally low levels of cortisol or they become less sensitive to cortisol. In either case, cortisol fails to restrain the inflammatory response. In one recent study of 72 women, depressed women had higher levels of IL-6 and TNF-α in response to an acute stressor than women who weren't depressed [15]. Also they were less sensitive to glucocorticoids that normally terminate the inflammatory response system [15]. The authors concluded that the depressed women had a blunted cortisol response to stress [15]. Groër and Morgan in their study of 200 postpartum women also noted a down-regulation of the HPA axis and abnormally low levels of cortisol in depressed women at 4 to 6 weeks postpartum [10].

#### (2) Depression, inflammation and preterm birth

Depression poses another health risk to puerperal women – increased risk of preterm birth. Several researchers have noted that depression and posttraumatic stress disorder increase risk of preterm birth, and inflammation may explain why [16,17]. In a study of 200 women at 4 to 6 weeks postpartum, depressed mothers had significantly smaller babies, more life stress, and more negative life events [10]. These women also had abnormally low cortisol levels meaning that the inflammatory response was not restrained. Another study in Goa, India (n = 270) found that babies of mothers who were depressed during their third trimester of pregnancy were significantly more likely to have low birth weight babies than their non-depressed counterparts. Mothers who were most severely depressed were at highest risk (Odds Ratio, OR: 2.5); these findings were significant even after for controlling for maternal age, maternal and paternal education, and paternal income [18].

In a prospective cohort study of 681 women, the rate of spontaneous preterm birth for depressed women was more than twice that of women who were not depressed (9.7% vs. 4%; OR: 3.3) [16]. The authors speculated on two possible pathways by which depression might lead to preterm birth. Depression can lead to elevated cortisol levels, which increases corticotrophin releasing hormone (CRH). CRH triggers parturition. The other possible mechanism they cited is activation of the proinflammatory cytokines and prostaglandin E2, which is secreted in response to cortisol and proinflammatory cytokines. Prostaglandin E2 plays a major role in uterine contractions [16].

The proinflammatory cytokines IL-6, IL-8, and TNF-α ripen the cervix before birth and these are also elevated when women are under stress. In a study of 30 pregnant women, Coussons-Read and colleagues found that TNF-α and IL-6 levels were significantly higher, and the anti-inflammatory cytokine IL-10 was significantly lower, in mothers who were stressed compared with mothers who weren't [4]. The authors hypothesized that inflammation was the likely mechanism to explain the relationship between maternal stress and preterm birth. They noted that high levels of inflammation (particularly IL-6 and TNF-α) were associated with preeclampsia and premature labor. Infection also increases the risk of preterm birth and TNF-α is released in response to both viral and bacterial infections. They concluded that high levels of proinflammatory cytokines may in fact endanger human pregnancies [4].

Inflammation may also explain another set of findings regarding preterm birth. In a study of 291 low-income pregnant women, participants were randomly assigned to receive either DHA-enriched eggs or regular eggs that they were to consume daily during the last trimester of their pregnancy [19]. This sample was predominantly African American (73%), a group generally at risk for preterm birth. DHA is a long-chain omega-3 fatty acid with anti-inflammatory effects (these effects are described in more detail in a subsequent section). Women who received the DHA-enriched eggs had an average increase in gestation of six days (SD = 2.3) [19]. The DHA-enriched eggs may have increased gestation length by decreasing inflammation.

#### (3) Physical and psychological stressors

As described in the previous section, human bodies are designed to respond a certain way when they are threatened. These threats can be physical or psychological; the physiological response is the same. Moreover, some types of stressors have both physical and psychological elements. Three stressors – sleep disturbance, pain, and trauma – are particularly relevant to new mothers. Studies that examine these stressors with regard to inflammation are described below.

##### (a) Sleep disturbances and fatigue

Sleep disturbances and fatigue are physical and psychological stressors that increase the risk of depression. Fatigue and sleep problems are often overlooked or minimized because they are so common in new mothers. But fatigue is often of great concern to mothers, and quantity and quality of sleep can alter stress both immune function and emotional well-being [9]. McEwen noted that short periods of disrupted sleep can wreck havoc on the physical health of even non-depressed people [20]. Disrupted sleep elevates evening cortisol levels (suggesting dysregulation of the HPA axis), increases glucose and insulin levels, and increases insulin resistance. And disturbed sleep is nearly universal in the postpartum period. In addition, mothers of premature babies or babies with high-needs temperaments may wake frequently at night well beyond the postpartum period. Psychological trauma and current stress can also compromise sleep quality [21,22]. All of these factors increase the risk of depression both directly and indirectly through sleep disturbance.

Sleep disturbance and depression are also mutually maintaining conditions: sleep disturbance can cause depression and depression causes sleep disturbances. Ross et al. noted that several factors suggest a relationship between sleep problems and depression in postpartum women [23]. These are as follows.

1) Insomnia is a significant risk for new-onset depression;

2) Sleep disturbances are common in most psychiatric disorders;

3) Treatments that manipulate sleep and circadian rhythms can be used to treat mood disorders.

In a review of polysomnographic studies of postpartum women, Ross et al. noted that there are differences in REM latency for women at risk for postpartum depression or who have current postpartum depression – reduced REM latency, increased total sleep time during pregnancy and decreased total sleep time postpartum [23]. REM latency refers to the time during the night when REM sleep becomes the predominant pattern. A pattern of reduced REM latency means that REM occurs earlier in the nightly sleep cycle, and is a symptom of depression. As a result of these sleep disturbances, women are more fatigued during the day. The authors noted that these changes may represent an underlying vulnerability to depression as they do with non-postpartum populations. They also noted that women with a history of affective disorders may be more sensitive to the normal physiologic changes of pregnancy.

Sleep disturbances and fatigue too are related to cytokine levels. When proinflammatory cytokine levels are high, fatigue increases. Moreover, the body experiences disturbed sleep as a physiological stressor [24]. In a sleep study of 22 patients with major depressive disorder (MDD) and 18 age-matched controls, inflammation was associated with sleep disturbances [25]. Prolonged sleep latency and REM density (two markers of disturbed sleep) were better predictors of IL-6 and ICAM (intercellular adhesion molecules – another inflammatory marker) than depressive symptoms. The authors concluded that sleep disturbances were at least the partial cause of elevated inflammation in depressed people [25].

Another study examined the role of the proinflammatory cytokine IL-1β in postpartum women [26]. The researchers found that higher levels of IL-1β were related to fatigue in women at four weeks postpartum. The authors speculated that IL-1β may have an indirect link to postpartum depression through fatigue [26]. Sleep deprivation is also a stressor that both activates the hypothalamic-pituitary-adrenal (HPA) axis and increases cytokine release in response [27].

In a study of women four to six weeks postpartum, Groër and colleagues found that mothers' fatigue levels correlated with their levels of stress and depression [27]. They also found that fatigue, stress and depression increased the risk of infection for both mother and baby. Fatigue, stress and depression make the immune system less effective, which increases the risk of infection. Interestingly, the same study also found that mothers who were stressed, depressed and fatigued had lower levels of prolactin in both their serum and milk. These same mothers also had higher levels of melatonin in their milk, the hormone that regulates circadian rhythms [27].

In a more recent study of 200 women at 4 to 6 weeks postpartum, Groër and Morgan found that depressed mothers reported more fatigue and daytime sleepiness than non-depressed mothers [10]. The depressed mothers had abnormally low levels of cortisol, which may also cause their fatigue. The authors describe how chronic fatigue syndrome, various chronic pain syndromes, and posttraumatic stress disorder are also associated with low cortisol levels. The depressed mothers also had more health problems since the baby was born and had more health-related events such as sprains, dental pain, and allergies. They had higher levels of perceived stress, anxiety and more negative life events. The serum IL-6 levels were three times higher in the depressed mothers, but this was not a significant difference because of measurement variability [10].

In summary, sleep disturbances and fatigue are physical stressors that increase the risk of depression. The relationship between sleep problems and proinflammatory cytokines appears to be bidirectional: sleep disturbances increase cytokines and cytokines increase sleep disturbance by delaying sleep onset, increasing daytime fatigue, and perpetuating the cycle of disturbed sleep and inflammation. Motivala and colleagues hypothesized that interventions that target disordered sleep may also lower inflammation, thereby lowering the risk of depression [25].

##### (b) Pain

Pain is another physical and psychological stressor related to increased inflammation and the risk of depression. Pain and depression are highly co-morbid conditions and may have a common etiology [28]. There are many types of pain that postpartum women are likely to experience. Pain can be the result of birth difficulties or breastfeeding difficulties. Pain can be caused by prior psychological trauma, which lowers the pain threshold so that normal sensations are perceived as painful [28]. Pain can also be caused by autoimmune disease that may appear for the first time in the postpartum period [1].

The relationship between pain and inflammation also appears to be bidirectional. When a woman experiences pain, stress hormones and levels of proinflammatory cytokines increase. High levels of proinflammatory cytokines, in turn, increase pain. Cytokines (especially IL-1) are stimulated by Substance P. Substance P is the neuropeptide that is high in patients with pain. In one study, patients with major depression or posttraumatic stress disorder were compared to healthy controls (n = 101) [30]. The patients with depression or PTSD had significantly elevated levels of Substance P in their cerebrospinal fluid. Moreover, the levels of Substance P rose significantly when the patients were presented with a laboratory-induced stressor that reminded them of their traumatic event. The authors concluded that Substance P was related to both depression and PTSD and responded to acute stress [30]. High levels of Substance P are also related to lower levels of serotonin, which increases the risk of depression. In addition, cytokines increase prostaglandin synthesis, including the prostaglandin cyclooxygenase-2 (COX-2), which increases pain [24,31-33]. In a study of mothers at eight weeks postpartum, a history of either violence or depression increased the risk of postpartum health problems including several types of postpartum pain [29].

Sleep disturbances can also increase pain. An example of the relationship between disrupted sleep and pain is in the chronic pain syndrome fibromyalgia. While this is a controversial diagnosis in some circles, researchers have noted that patients with fibromyalgia have a number of physiologic abnormalities that cannot be influenced by patient report. These include increases in Substance P (up to three times as much compared to healthy controls), decreases in serotonin and the serotonin metabolite 5-HIAA in the cerebral spinal fluid, abnormal brain activation patterns, and of relevance to the present discussion, abnormal sleep patterns [28]. A pattern frequently found in patients with fibromyalgia is interrupted delta (slow-wave) sleep. Interrupted delta sleep means that patients' bodies do not make repairs to muscular micro-traumas that have occurred during the day. The net result is all-over body pain [34]. Fibromyalgia has even been induced in the laboratory, where patients' sleep was monitored, and every time they went into delta sleep they were woken up. By the next morning, they had head-to-toe pain, which resolved once they had a normal night's sleep [34].

An interesting exception to the pain-sleep disturbance phenomenon is co-sleeping. Polysomnographic studies of breastfeeding mothers who are sleeping next to their babies for all or part of the night indicate that the mothers spend less time in deep sleep than mothers who are not co-sleeping. Despite significantly decreased slow-wave sleep, co-sleeping mothers do not appear to report an increase in body pain [35]. Co-sleeping may be less stressful for mothers than needing to completely wake for night time feeding. Or the higher levels of prolactin that co-sleeping mothers have may have an impact decrease inflammation and pain. This issue has never been specifically addressed and would be an interesting topic for future research.

Pain can be a potent trigger for depression in postpartum women. A common type of pain in the first few weeks after birth is nipple pain. A study of 113 breastfeeding women (48 with nipple pain, 65 without) demonstrated that women with pain were significantly more likely to be depressed than women without pain (38% vs. 14%) [36]. Women in the pain group also had significantly higher scores on the Profile of Mood States questionnaire. Once the pain resolved, the scores on these scales dropped to normal levels [36].

Unfortunately, nipple pain appears to be common, even among educated, middle-class women – the group most likely to breastfeed. In one study in Minneapolis, Minnesota, an astonishing 50% of women had nipple pain at five weeks [37]. Another study from Toronto, Canada had similar results; 52% of mothers reported cracked or sore nipples at two months postpartum [29].

In summary, postpartum pain is a common experience among women who have recently given birth [29]. Addressing pain promptly, and providing mothers the means to cope with their pain can halt the cascade of stress hormones and proinflammatory cytokines, decreasing their risk of depression.

##### (c) Current trauma or history of trauma

Psychological trauma can also have an impact on depression and cytokine levels. Like depression, posttraumatic stress disorder (PTSD) is a dysregulation of a normal stress response. Cortisol levels can be abnormally high or low. Previous studies found that people with PTSD had abnormally low cortisol levels. When cortisol is not there to inhibit the inflammatory response system, people who have PTSD have increased cytokine activity [38-40]. Even if cortisol is elevated, the receptors can be less sensitive to cortisol and fail to restrain the inflammatory response [41,42].

According to the *Diagnostic and Statistical Manual *[43], a traumatic event is one in which the person felt that death or serious injury was possible for themselves or a loved one, and the person responded with fear, helplessness or horror. In addition, there must be symptoms in each of these three clusters: 1) intrusion: frequent re-experiencing of the event via nightmares or intrusive thoughts, 2) avoidance: numbing or lack of responsiveness to or avoidance of current events that remind patients of their trauma, and 3) hyperarousal: persistent symptoms of increased arousal including jumpiness, sleep disturbances or poor concentration [43].

PTSD can be caused by a pre-existing trauma, such as sexual assault or natural disaster. Or it can be caused by the birth itself. In a review of the literature, Beck found that the rates of women who met full criteria for PTSD following birth ranged from 1.5% to 6% [44]. The study with 1.5% excluded women with prior depression or PTSD – the very women who are most vulnerable. Even the highest percentage (6%) may seem relatively small. By way of comparison, in the weeks following the September 11th terrorist attacks in 2001, 7.5% of residents of lower Manhattan living near Ground Zero met full criteria for PTSD [45]. It's shocking to realize that the percentage of women meeting full criteria for PTSD after birth is not substantially different than the rates following a terrorist attack. Moreover, even if women do not meet full criteria, they often have symptoms and these can be troublesome, interfere with their sleep and increase the risk of depression [1,46].

###### 1) The impact of highly stressful births on breastfeeding

Women may also have experiences that are highly stressful, while not leading to PTSD [28], can cause breastfeeding difficulties. Women who experience highly stressful births are likely to have abnormally high cortisol levels in the first few days postpartum. And elevated cortisol levels may cause problems. In a study from Guatemala, researchers measured the cortisol levels of 136 women before or after birth. For women with the highest levels of cortisol, lactogenesis II (the onset of copious milk supply) was delayed for several days [47].

###### 2) Prior history of depression or trauma

A woman with a history of depression or trauma is often more vulnerable to current life stresses. One way that that vulnerability manifests is a more rapid inflammatory response to current stressors. This effect has been noted in both human and animal research. For example, in an animal study, prior exposure to a stressor (in this case, inescapable foot shock) led to a hypersensitivity of the inflammatory response system and more rapid release of proinflammatory cytokines when exposed to a subsequent stressor [48]. This is consistent with human studies of childhood abuse that indicate that men and women who are abused in childhood have a significantly increased vulnerability when exposed to current life stressors. They may respond to these current situations with either depression of PTSD [39].

Kiecolt-Glaser and colleagues also noted that stress and depression appear to prime the inflammatory response so that it is more reactive to subsequent stressors [5]. During the postpartum period, women experience a number of significant stressors, such a sleep deprivation and postpartum pain, that increase inflammation and the subsequent risk of depression [2,5]. Women with prior histories of severe stress, depression and trauma are at increased risk of postpartum depression in part because of the way their bodies are primed to react to stress.

### Implications: goals of prevention and treatment

A PNI approach suggests two important and related goals for the prevention and treatment of depression in new mothers: reduce maternal stress and reduce maternal inflammation. These recommendations are described below.

#### (1) Reduce maternal stress

Since physical and psychological stressors trigger the inflammatory response system, the first goal for preventing or treating depression is to reduce maternal stress. And one important way to do this is for health care providers to encourage mothers to breastfeed, and to support ongoing breastfeeding relationships.

##### (a) The adaptiveness of breastfeeding

When breastfeeding is going well, it protects mothers from stress [8,9], thereby protecting maternal mood. A study of 28 mothers who were both breast- and bottle-feeding measured mothers' stress levels immediately before and after both types of feeding. This study was a major methodological improvement over previous studies in that women served as their own controls. Since there were not pre-existing differences between the groups, it was possible to attribute the observed difference in mood to feeding method alone. The researchers found that breastfeeding decreased negative mood and bottle feeding decreased in positive mood in the same women [49].

Another study compared stress levels of three groups of women: women who were exclusively breastfeeding (n = 84), women who were exclusively feeding infant formula (n = 99), and non-postpartum healthy volunteers (n = 33) [50]. The researcher found that breastfeeding women had lower perceived stress, depression and anger, and more positive life events than the controls. Serum prolactin was inversely related to stress and mood in formula-feeding mothers, but this was not true for the breastfeeding mothers. The author concluded that breastfeeding appears to be mildly protective of maternal mood [50]. More recently, these same researchers found in a study of 200 women at 4–6 weeks postpartum, that depressed women were significantly less likely to be breastfeeding and they had significantly lower serum prolactin levels. They also reported significantly more life stress and anxiety [10].

A study of 43 breastfeeding women found that both breastfeeding and holding their babies without breastfeeding significantly decreased ACTH, plasma cortisol, and salivary free cortisol [51]. In response to an induced stressor, cortisol responses were attenuated in breastfeeding women for a short time after feeding their babies. The authors concluded that suckling, but not breastfeeding in general, provided a short-term suppression of the stress-related cortisol response and HPA axis response to mental stress [51]. They argued that this short-term suppression provided several evolutionary and biological advantages. It isolated the mother from distracting stimuli, facilitated the women's immune system, protected the babies from high cortisol in the milk and prevented stress-related inhibition of lactation. Based on their review, Groër, Davis and Hemphill drew similar conclusions [9]. They noted that the neuroendocrinology of breastfeeding women possibly down-regulated the stress response. This down-regulation protects the breastfeeding mother and directed her toward milk production, conservation of energy, and nurturing behaviors.

Exclusive breastfeeding also increases the effectiveness of the mothers' immune system [50,51]. In a study of 181 women at 4 – 6 weeks postpartum, perceived stress, depression, anxiety, anger and negative life events were related to decreased immune competence for the formula-feeding mothers [52]. This relationship was not present in the breastfeeding mothers who were protected from the harmful effects of stress on immunity [52].

###### Breastfeeding and stress in babies

Breastfeeding not only reduces stress for mothers; it also lowers stress that babies experience when their mothers are depressed. Jones et al. compared the infants of four groups of women: depressed mothers who were either breast or bottle feeding, and non-depressed mothers who were either breast or bottle feeding [53]. The outcome was the babies' electroencephalogram (EEG) patterns. This measure is used to determine if infants have physiological symptoms of depression – in this case, a pattern of right frontal asymmetry. Right frontal asymmetry is a pattern that also is found in chronically depressed adults.

The researchers found that the babies of the depressed/non-breastfeeding mothers had the abnormal pattern of right-frontal asymmetry [53]. In contrast, infants of the depressed/breastfeeding mothers had normal EEG patterns. In other words, breastfeeding protected these babies from the effects of maternal depression. The authors explained their findings by noting that the depressed/breastfeeding mothers did not disengage from their babies the way the depressed/bottle-feeding mothers did. The depressed/breastfeeding mothers continued to look at, touch and stroke their babies because these behaviors are built into the breastfeeding relationship. In contrast, when a mother bottle feeds, she doesn't have to even hold her baby. So it is easier for her to disengage, leading to the symptoms that babies typically exhibit when their mothers are depressed [53].

In summary, breastfeeding protects maternal mood by lowering stress. When stress levels are lower, the mother's inflammatory response system will not be activated, thereby lowering her risk of depression. However positive these results, I must issue one caveat: they only apply when breastfeeding is going well. As noted earlier, when breastfeeding that is not going well, particularly if there is pain, it becomes a trigger to depression rather than something that lessens the risk. Mothers' mental health is yet another reason to intervene quickly when breastfeeding difficulties arise.

##### (b) Exercise and depression

Another way mothers can reduce stress is to exercise, which has also been found to alleviate depression. In a Finnish population study (n = 3,403), men and women who exercised two to three times a week experienced significantly less depression, anger, cynical distrust, and stress than men and women who exercised less frequently [54].

The efficacy of exercise was also demonstrated in a randomized trial of patients with major depressive disorder. In this study, 156 patients with major depression (>50 years old) were randomized into one of three treatment groups: aerobic exercise alone, sertraline (antidepressant) alone, and a combination of exercise and sertraline [55]. After four months, all three groups showed improvement and there were no significant difference between the groups. In other words, those who were in the exercise-only group experienced as much recovery as those who took either medication alone or in combination with exercise. The more striking findings, however, occurred at 10 months. At that time, the exercise-only group had a significantly lower rate of relapse than either the medication alone or medication/exercise groups. The authors speculated that this was because by learning to exercise, the study had given the exercise-only group a coping tool that they could use when faced with life stressors [55]. This study is particularly noteworthy because it was the first to try exercise as a treatment for major depression.

In summary, by linking stress and depression, the PNI framework provides a rationale for why exercise would be an effective intervention for depression – something practitioners can share with mothers. The goal is improved mental health, not weight loss (although some might occur). Depressed mothers need this information because exercise is often the very last thing they feel like doing. But exercise is an effective technique for both decreasing their risk of depression and helping them cope with the stresses and strains of early motherhood.

#### (2) Reduce inflammation

The second recommendation is for mothers to reduce inflammation. As described earlier, psychosocial and physical risk factors for depression increase inflammation, which increases depression risk. One way to decrease inflammation is through consumption of long-chain omega-3 fatty acids. Omega-3s show promise in the treatment of mood disorders according to a 2006 expert panel convened by the American Psychiatric Association [56]. And they do so, in part, by decreasing inflammation. These studies and their relevance to pregnant and postpartum women are described below.

##### (a) Long-chain omega-3 fatty acids and depression

Over the past 100 years, the diet of people in many Western cultures has changed substantially. Some have theorized that these changes could explain the increase in depression during this same time period [2,5,57]. Of particular concern is the change in the ratio of omega-6:omega-3 fatty acids, with dramatically decreased omega-3s and increased omega-6s. Omega-6 fatty acids are found in vegetable oils and are a staple of processed foods. Omega-3s are found in fatty fish and some plant sources. While some omega-6s are necessary for good nutrition, they become harmful when the ratio of omega-6s to omega-3s is too high – as it is in modern diets. Kiecolt-Glaser and colleagues noted that the hunter-gatherer diet had an estimated ratio of omega-6s:omega-3s of 2:1 or 3:1 [5]. The typical North American diet ranges from 15:1 to 17:1. In Australia and New Zealand the ratio is 10:1 [57]. With regard to depression, the long-chain omega-3s are of interest: EPA and DHA [58]. ALA is the omega-3 found in flax and other plant sources. ALA is an essential fatty acid, and while important for good nutrition, it has no efficacy in the prevention or treatment of depression [58].

###### (i) Anti-inflammatory activity of omega-3s

Omega-3s are powerful anti-inflammatories and lower proinflammatory cytokines. In a large population study, high levels of omega-3s (ALA, EPA and DHA) were related to lower levels of the proinflammatory cytokines IL-1α, IL-1β, IL-6, and TNF-α and higher levels of anti-inflammatory cytokines such as IL-10. For people with low levels of omega-3s, the opposite was true: these people had high levels of proinflammatory cytokines and low levels of anti-inflammatory cytokines [59]. Higher levels of proinflammatory cytokines increase the risk for depression.

In a study of older adults, the combination of depression and higher omega-6: omega-3 ratios dramatically increased levels of proinflammatory cytokines (IL-6 & TNF-α) beyond the individual contribution of either variable alone. The authors noted that a diet with a high ratio of omega-6s:omega-3s increases the risk of both depression and diseases related to chronic inflammation including heart disease, diabetes and cancer [5].

EPA and DHA may also increase resilience to stress by regulating and attenuating the stress response. Maes and colleagues noted that an imbalance of omega-6s:omega-3s leads to an overproduction of proinflammatory cytokines in response to psychological stress [11]. When omega-3 levels are higher, the levels of proinflammatory cytokines are reduced. Their study included 27 college students who were exposed to a laboratory-induced stressor. The students with lower serum levels of omega-3s had a significantly greater stress-induced elevation of TNF-α and IFN-γ than those with higher serum omega-3s or a lower ratio of omega-6s:omega-3s [11].

###### (ii) Omega-3s in population studies

Population studies have provided support for the efficacy of omega-3s in preventing depression and other mental health problems by demonstrating that populations who naturally consume more omega-3s in their diets (usually by eating fatty coldwater fish, e.g. such as salmon or mackerel) have lower rates of a variety of mental health problems. For example, populations with higher levels of EPA and DHA in their diets had lower levels of major depression, bipolar disorder, and future suicide risk [60-62]. A prospective study in Osaka, Japan of 865 pregnant women, however, failed to find a relationship between fish intake or the intake of omega-3 fatty acids and prevention of postpartum depression [63]. These findings could be due to a ceiling effect in that the entire population already eats large amounts of seafood [63].

###### (iii) Treatment with EPA and DHA

Positive findings have been noted in randomized clinical trials, where researchers have given either EPA/DHA supplements or a placebo to people currently receiving treatment for unipolar or bipolar depression. When EPA was added to patients' normal regimen of antidepressants, EPA made the antidepressants more effective in treating depression than the placebo [64]. Similarly, in a study of childhood depression, children who received EPA and DHA in addition to their medication had significantly improved depression compared with children who received their medication and a placebo [65]. EPA also helped stabilize symptoms of bipolar disorder in a 12-week double-blind trial [66].

In a recent review, EPA had efficacy in the treatment of depression in four of the six studies reviewed [67]. The authors found that 1 gram of EPA per day was the effective dose. Doses higher than two grams seemed to have the reverse effect [67]. EPA affects the function of both the proinflammatory cytokines and the eicosanoids. The eicosanoids include prostaglandins, leukotrienes and thromboxanes [57]. Given its direct action on proinflammatory cytokines, the efficacy of EPA as a treatment for depression is consistent with previous studies on depression and inflammation.

A preliminary study demonstrated that EPA and DHA may have efficacy in the treatment of postpartum depression. The study was limited, however, by a small sample, no control group, and subjects who were not blind to the treatment condition. This study was intriguing, however, because depression decreased 48% to 51% in the eight-week study period [6]. At the very least, these findings suggest that a follow-up study would be useful.

###### (iv) DHA in the perinatal period

During the perinatal period, DHA appears to be particularly important [68]. Pregnant women's diets are often deficient in DHA, which is unfortunate given babies' high need for it in utero. And this may put their mothers at risk for depression. As Rees and colleagues describe, during the last trimester of pregnancy, babies accumulate an average of 67 mg/day of DHA [57]. The average intake for Australian mothers is 15 mg/day. In contrast, DHA consumption among Japanese, Koreans, and Norwegians is about 1000 mg/day. Because babies need DHA for brain and vision development, women's bodies will preferentially divert DHA to the baby, and the baby will take the DHA it needs from maternal stores. With each subsequent pregnancy, mothers are further depleted [36,57]. Current recommended intake is 200 to 400 mg/day as a minimum dose.

DHA appears to have a role in the prevention of depression, but according to two recent reviews, has no efficacy in treatment of depression when used alone [67,69]. In the Adelaide Mothers' and Babies' Iron Trial, a 1% increase in plasma DHA was related to a 59% decrease in depressive symptoms postpartum [57]. In a large population study, women who consumed high amounts of seafood during pregnancy and had high levels of DHA in the milk had lower rates of postpartum depression [70].

DHA may have another effect that could help prevent depression postpartum. In a study of infant sleep, mothers with high levels of DHA during pregnancy had babies who exhibited a more mature sleep pattern in the first few days of life [71]. The investigators examined the ratio of quiet to active sleep using a monitor placed beneath the crib mattress. A higher percentage of quiet sleep is characteristic of older babies, and is considered a more mature sleep pattern. Babies whose mothers had high levels of DHA during pregnancy exhibited more mature sleep patterns as neonates. The investigators concluded that babies of high-DHA mothers had more mature central nervous systems than babies of mothers who were low in DHA. Although this was not study of depression per se, babies with more mature sleep patterns also allow mothers to get more uninterrupted sleep – and this could have an indirect effect on their mothers' mental health [71].

###### (v) Sources of EPA and DHA

As the previously cited studies indicate, women are often deficient in long-chain omega-3 fatty acids during the perinatal period. However, pregnant or breastfeeding women often need to limit the amount of fish they eat, the prime source of EPA and DHA, because of contaminants in seafood. Fortunately, there are a alternative sources of EPA and DHA that are tested for contaminants and are contaminant free. A listing of sources of EPA/DHA that are tested for contaminants can be seen in Additional file 1.

##### (b) Other anti-inflammatory treatments for depression

In addition to omega-3s, several standard treatments for depression are also anti-inflammatory. For example, the herbal antidepressant St. John's wort has long been known to be anti-inflammatory [72]. Traditional antidepressants are also anti-inflammatory and this may explain their efficacy. For example, a recent study compared C-reactive protein levels in cardiac patients with major depression before and after treatment with selective serotonin reuptake inhibitors (SSRIs). In these patients, C-reactive protein dropped significantly after treatment, independent of whether depression resolved [73].

One could even argue that cognitive therapy is anti-inflammatory. Two recent studies have demonstrated that negative beliefs, such as hostility, can increase the levels of proinflammatory cytokines – especially IL-6 [74,75]. Cognitive therapy is a treatment for depression with known efficacy [76]. The primary goal of cognitive therapy is to reduce negative cognitions. Since negative cognitions increase inflammation, reducing their occurrence will have physical effects as well – primarily reducing inflammation.

## Conclusion

Recent research has identified inflammation as a key factor in depression, and inflammatory response system is triggered by both physical and psychological stress. Postpartum women are particularly at risk because their inflammation levels are naturally elevated in the last trimester of pregnancy, and this elevation continues through the postpartum period. Two approaches may prevent depression or reduce its severity: lowering maternal stress and reducing inflammation. Breastfeeding has been shown to reduce stress and protect maternal mood. Breastfeeding also reduces stress of babies of depressed mothers and protects them from the harmful effects of maternal depression. Treatment approaches that are anti-inflammatory have efficacy in treating depression. These include EPA and DHA, exercise, cognitive therapy, herbal anti-depressants such as St. John's wort, and standard antidepressants. Research is needed to assess if anti-inflammatory approaches used proactively can prevent depression from occurring in the first place.

## Abbreviations

ALA: α-linolenic acid

COX-2: cyclooxygenase-2

CRP: C-reactive protein

DHA: docosahexaenoic acid

EEG: electroencephalogram

EPA: eicosapentaenoic acid

HPA: hypothalamic-pituitary-adrenal axis

ICAM: intercellular adhesion molecule

IL-1β: Interleukin-1beta

IL-6: Interleukin-6

IL-10: Interleukin-10

IFN-γ: Interferon-γ

IRS: inflammatory response system

PNI: psychoneuroimmunology

PTSD: posttraumatic stress disorder

SSRI: selective serotonin reuptake inhibitor

TNF-α:Tumor Necrosis Factor-alpha

## Competing interests

The author worked briefly in 2006 as a consultant and continuing education provider for Martek Biosciences Corporation. This paper was written independent of that work and was completed several months after the consulting agreement ended.

## Supplementary Material

Additional file 1 Sources of EPA/DHA that are tested for contaminants Click here for file
