# Supplementary material for: A new paradigm for depression in new mothers: the central role of inflammation and how breastfeeding and anti-inflammatory treatments protect maternal mental health
Source: Int Breastfeed J. 2007 Mar 30;2:6. doi: 10.1186/1746-4358-2-6 (PMC1855049; doi:10.1186/1746-4358-2-6)
Supplement: Additional file 1 — Sources of EPA/DHA that are tested for contaminants [file 1746-4358-2-6-S1.doc]

Table 1

Sources of EPA/DHA that are tested for contaminants

**Pharmaceutical-Grade Fish Oil (EPA & DHA)**

##### Carlson Labs ([www.CarlsonLabs.com](http://www.carlsonLabs.com/))

##### Vital Nutrients ([www.VitalNutrients.net](http://www.vitalnutrients.net/vn.asp))

##### Brands of over-the-counter fish-oil supplements verified by the U.S. Pharmacopeia ([www.usp.org](http://www.usp.org/USPVerified/dietarySupplements/))

**Berkley & Jensen, Equaline, Kirkland Signature, Nature Made, NutriPlus**

**Vegetarian DHA Supplements**

**Nature’s Way DHA (**[**www.NaturesWay.com**](http://www.naturesway.com/NaturesWay/products.aspx?hero=1&maxcols=1&maxitems=10&productid=Microalgae_Oil_Sub_Category)**)**

**O-mega-Zen-3 (**[**www.Nutru.com**](http://www.nutru.com/)**)**

**Prescription prenatal supplements with DHA**

**OptiNate (**[**First Horizons Pharmaceutical**](http://www.prenate.com/optinate)**)**

**Citracal Prenatal + DHA (**[**Mission Pharmacal**](http://www.citracalprenatalrx.com/)**)**

**DHA-Fortified Foods**

**DHA-fortified eggs (**[**Gold Circle Farms**](http://www.goldcirclefarms.com/)**)**

[**Oh Mama!**](http://www.ohmamabar.com/) **Nutrition bar for pregnant and breastfeeding women**

[**Odwalla Soymilk**](http://www.odwalla.com/product1.asp?p=soymilk&sw=1)

**Belly Bar (**[**Nutrabella**](http://www.nutrabella.com/)**)**

The author has no financial connection with any of these companies or products.
